# Selective amygdalohippocampectomy via the paramedian supracerebellar-transtentorial approach for mediobasal temporal epilepsy

**DOI:** 10.3171/2024.4.FOCVID2447

**Published:** 2024-07-01

**Authors:** Yücel Doğruel, Serdar Rahmanov, Abuzer Güngör, Uğur Türe

**Affiliations:** 1 Department of Neurosurgery, Yeditepe University School of Medicine, İstanbul, Türkiye;; 2 Department of Neurosurgery, Tunceli State Hospital, Tunceli, Türkiye; and; 3 Department of Neurosurgery, Istinye University, Faculty of Medicine, İstanbul, Türkiye

**Keywords:** amygdalohippocampectomy, epilepsy surgery, neurocysticercosis, seizure, transtentorial

## Abstract

Selective amygdalohippocampectomy via the pterional transsylvian approach is a feasible option for many patients with mediobasal temporal epilepsy. However, it may be insufficient for patients when the posterior hippocampal region is involved. The paramedian supracerebellar transtentorial approach offers precise anatomical orientation when exposing the entire length of the mediobasal temporal region, including the fusiform gyrus. In addition, this approach allows selective amygdalohippocampectomy without any neocortical damage. This video presents the successful treatment of a patient with posterior hippocampal sclerosis and mediobasal temporal epilepsy through the paramedian supracerebellar transtentorial approach.

The video can be found here: https://stream.cadmore.media/r10.3171/2024.4.FOCVID2447

## Transcript

### 0:20 Introduction.

This video demonstrates selective amygdalohippocampectomy via the paramedian supracerebellar-transtentorial approach for a patient with mediobasal temporal epilepsy.

### 0:33 Clinical Presentation.

A 45-year-old female presented with a 10-year history of drug-resistant epilepsy.

She experienced limbic seizures 8 to 10 times daily and a secondary generalized seizure once every 2 to 3 months. Two years prior to the onset of seizures, the patient had an extended visit to South Asia.

### 0:57 Neuroimaging Findings.

Previous radiological images from other clinics showed no lesions. A new MRI revealed a pathological T2 signal increase in the body and tail of the left hippocampus, extending to the amygdala. It also revealed a lesion in the left posterior hippocampal region. The lesion is marked with yellow arrowheads.

The patient underwent video-EEG monitoring. The ictal video-EEG indicated mediobasal temporal epilepsy.

### 1:30 Approach Selection.

There are several alternative options for patients with mediobasal temporal epilepsy. These are anterior temporal lobectomy with amygdalohippocampectomy,^1^ anteromedial temporal lobectomy with amygdalohippocampectomy,^2^ lateral transcortical selective amygdalohippocampectomy,^3^ and pterional transsylvian selective amygdalohippocampectomy.^4^

We did not choose the first three alternatives because they involve cortical injury, and we did not choose the last alternative due to the posterior hippocampal extension of the pathology.

We decided to perform selective amygdalohippocampectomy via the paramedian supracerebellar transtentorial approach, which was described by our team, as it allows us to reach the entire mediobasal temporal region without involving any cortical injury.^5^

### 2:36 Positioning.

The patient is positioned semisitting, with the body elevated at a 20° angle. Larger angles elevate the risk of venous air embolism.^6^

### 2:46 Surgical Opening.

A left-sided paramedian linear skin incision is made, and a paramedian suboccipital craniotomy is carried out.^5^

A 15-mm transverse dural incision is made at the lower aspect of the craniotomy. This allows access to the cisterna magna and releases cerebrospinal fluid. Then, a curvilinear opening in the dura is made about 15 mm below the transverse sinus.

The dura is suspended superiorly, and the supracerebellar surface of the tentorium is inspected with a particular focus on venous variations. The patient has a tentorial sinus, indicating a cerebellar hemispheric tentorial bridging vein located lateral to the surgical route. This vein and sinus should be preserved to prevent complications.^7^

The tentorium is coagulated and subsequently cut through the tentorial hiatus and the petrous ridge, respectively. The cerebellar hemispheric tentorial bridging vein and its draining sinus are preserved.

The tentorium is retracted with three hanging sutures. This maneuver exposes the inferior surface of the left mediobasal temporal region.

### 4:17 Intraoperative EEG.

A strip electrode is placed on the surface of the parahippocampal gyrus, and intraoperative EEG is done to define the epileptogenic area. The intraoperative EEG demonstrates epileptic activity. The strip electrode is removed and the arachnoid of the ambient cistern is dissected.

### 4:36 Surgical Technique.

A perpendicular posterior parahippocampal incision is made with bipolar forceps at the level of the colliculi. It is limited laterally by the collateral sulcus.

The subiculum is dissected with subpial resection. Careful attention is paid to preserving the major inferior temporal arteries originating from the P2 and P3 segments of the posterior cerebral artery. After complete dissection of the subiculum, the left thalamus is identified.

The temporal horn of the lateral ventricle is identified deep within the collateral sulcus on the lateral border of the dissection. Identifying the temporal horn is the most important step of the surgery, as it indicates the lateral border of the dissection. The posterior portion of the left parahippocampal gyrus is dissected and removed. The dissection proceeds and the remaining part of the posterior hippocampus is resected.

A solid lesion is observed in the resected portion of the parahippocampal gyrus and collected for histopathologic examination. Then, the body of the hippocampus is dissected from the ventricular surface, and the roof of the temporal horn is identified. The hippocampal artery is coagulated and cut. Then, the uncal artery is coagulated and cut, and the uncus with the band of Giacomini are dissected from the ambient cistern. The body of the hippocampus is dissected. After circumferential dissection, it is removed and collected for histopathologic examination.

The amygdala is identified. Resection of the amygdala is done carefully during subpial removal of its medial aspect, as both the optic tract and the anterior choroidal artery are at risk. The amygdala is dissected circumferentially and collected for histopathologic examination along with the head of the hippocampus. Residual hippocampus tissue is dissected with suction, and then resection is completed.

A neuro-endoscope is introduced to visualize the surgical cavity. Endoscopic visualization confirms the selective amygdalohippocampectomy. The surgical cavity is irrigated with saline solution, and hemostasis is achieved.

### 8:36 Closure.

Finally, the dural incisions are closed in watertight fashion.

### 8:42 Outcome.

The postoperative MRI confirmed the left-sided selective amygdalohippocampectomy and complete resection of the lesion. The postoperative visual field test showed no visual field defects. The patient was discharged without complications.

Histopathologic examination revealed a calcified neurocysticercosis cyst, which is not a common diagnosis in our country, along with hippocampal sclerosis. Because of the calcification, antiparasitic treatment was not administered to the patient.^8^

After surgery, the patient’s medications were gradually reduced and eventually discontinued. The patient is in the 10th year of follow-up, with no seizures reported.

### 9:29 Conclusion.

The paramedian supracerebellar transtentorial approach is well suited for selective amygdalohippocampectomy, as it is guided by anatomical landmarks.^5^

This approach allows especially the posterior portion of the hippocampus and parahippocampal gyrus to be safely removed, potentially leading to improved seizure control, particularly in patients with posterior hippocampal sclerosis. One advantage of this approach is eliminating the risk of injury to major white matter structures. Additionally, it prevents potential injury to superficial and deep sylvian veins, the middle cerebral artery and its branches, and especially the temporal pole and temporal neocortex.

Thank you.

## Acknowledgments

We thank Aylin Akçaoğlu for artistic illustrations and Julie Yamamoto and Daniel Yamamoto for editorial contributions to the text.

## Disclosures

The authors report no conflict of interest concerning the materials or methods used in this study or the findings specified in this publication.

## Author Contributions

Primary surgeon: Türe. Assistant surgeon: Doğruel, Güngör. Editing and drafting the video and abstract: all authors. Critically revising the work: Türe, Doğruel, Güngör. Reviewed submitted version of the work: Türe, Doğruel, Güngör. Approved the final version of the work on behalf of all authors: Türe. Supervision: Türe.

## Supplemental Information

### Patient Informed Consent

The necessary patient informed consent was obtained in this study.
